# Topological States Characterized by Mirror Winding Numbers in Graphene with Bond Modulation

**DOI:** 10.1038/s41598-017-16334-0

**Published:** 2017-11-28

**Authors:** Toshikaze Kariyado, Xiao Hu

**Affiliations:** International Center for Materials Nanoarchitectonics (WPI-MANA), National Institute for Materials Science, Tsukuba, 305-0044 Japan

## Abstract

Localized electrons appear at the zigzag-shaped edge of graphene due to quantum interference. Here we propose a way for harnessing the edge electronic states to make them mobile, by incorporating a topological view point. The manipulation required is to introduce a pattern of strong-weak bonds between neighboring carbon atoms, and to put side by side two graphene sheets with strong-weak alternation conjugating to each other. The electrons with up and down *pseudospins* propagate in opposite directions at the interface, similar to the prominent quantum spin Hall effect. The system is characterized by a topological index, the mirror winding number, with its root lying in the Su-Schrieffer-Heeger model for polymer. Taking this point of view, one is rewarded by several ways for decorating graphene edge which result in similar mobile electronic states with topological protection. This work demonstrates that celebrated nanotechnology can be used to derive topological states.

## Introduction

Graphene exhibits linear dispersions in its energy spectrum originated from the honeycomb structure^1^, which encapsulates relativistic physics expected in high energy into the two-dimensional material^2^ and gives birth to many intriguing properties such as high electron mobility, heat conductance, and the chiral quantum Hall effects under magnetic field. For device applications, however, it is desired to turn graphene into a semiconductor by opening a controllable energy gap in the linear dispersion, which in a physics language is equivalent to attaching a mass to electron governed by the Dirac equation^2–4^. Noticeably, the emergent mass term has also played a key role in the development of the topological phases of matter. Haldane was the first to recognize that, in terms of complex next-nearest-neighbor (nnn) hopping integral, a valley-dependent mass can be induced in the massless Dirac-like dispersion in honeycomb structure, resulting in a quantum anomalous Hall effect^5^. Kane and Mele then noticed that, taking into account the spin degree of freedom of electron, the spin-orbit coupling (SOC) yields naturally two copies of the Haldane system in the spin-up and -down channels related to each other by time-reversal (TR) operation^6,7^, which is known as a quantum spin Hall effect (QSHE).

**Figure 1:**
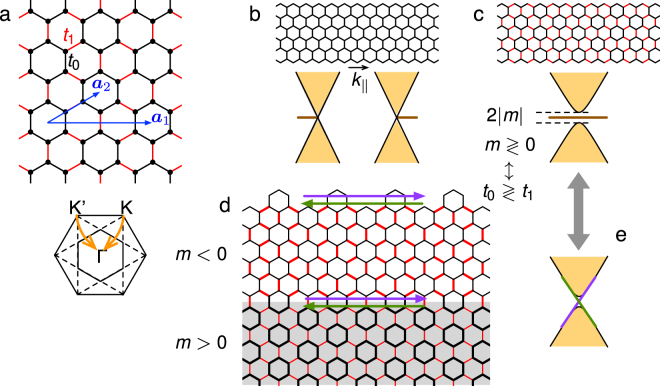
(**a**) The tight-binding model on honeycomb lattice with modulated nearest-neighbor hopping integrals with two unit vectors ***a***_1,2_ used throughout the paper. The schematic picture of the original and the folded Brillouin zone is also shown. (**b**) and (**c**) Schematic pictures of the edge band structure for the ribbon of zigzag edges without and with hopping modulation. (**d**) Schematic pictures for decorated edge and interface, and (**e**) the dispersive edge states.

Nearest-neighbor (nn) hopping integral only can also open a gap in the Dirac-like linear dispersions in honeycomb lattice: due to the nesting effect caused by hopping texture over hexagons^8^, the Dirac cones at K and K’ points at the corners of Brillouin zone of ambient graphene are folded to Γ point (see Fig. 1a) and meanwhile an energy gap is opened. It was argued that this operation yields a quantum *pseudospin* Hall effect, where the pseudospin is associated with the orbital angular momentum accommodated on the hexagon, in contrary to other approaches which compose pseudospin in terms of sublattice and/or valley in honeycomb structure. Remarkably, because the mass induced by the hopping texture is given by *m* = *t*_1_−*t*_0_ (see Fig. 1), there appears an interface state with topological protection when two patches with opposite masses are attached to each other (Fig. 1d).

It is worthy noticing that the interface states thus derived transport unidirectional currents, in contrary to the immobile electronic state at the zigzag edge of graphene ribbon^9^ (Fig. 1b). This difference can be best captured by the topological index called mirror winding number. For simplicity we consider the case that the nnn hopping is totally absent (as well as other long-range hoppings). Exploring this sublattice symmetry (also known as chiral symmetry) in a graphene ribbon with zigzag edge, we can prove that electronic wave functions in the subspaces of the even and odd parity with respect to mirror operation along a plane normal to the zigzag edge are characterized by winding numbers (*n*_+_, *n*_−_) as (1, 0)/(0, 1) for *m* > 0/*m* < 0 respectively. Bringing the two insulators side by side (see Fig. 1d) results in the difference in mirror winding numbers (1, −1), which renders a zero total winding number, and thus the in-gap interfacial states dispersive and able to transport currents. In contrast, at the interface of each of the two insulators to vacuum, the two pairs of mirror winding numbers (1, 0) and (0, 1) yield a nonzero winding number in both cases, which is responsible for the well-known flat-band and static edge state at the zigzag edge of graphene ribbon.

Real-space textures in the hopping energy can be realized in systems of honeycomb structure in terms of cutting-edge technologies of material preparation. In the molecular graphene achieved on the Cu [111] surface, a pattern in hopping integrals was realized by putting extra CO molecules, which induces an energy gap of ~100 meV in the otherwise Dirac dispersion^10,11^, where the system geometry (including the edge shape, which will be important in the latter arguments) can be manipulated accurately by the advanced STM technique. Or, simply putting a graphene sample on an appropriate substrate possibly causes the hopping patterns^12^. Turning to metamaterials which are analogs to materials with ions replaced by periodic arrangements of dielectric constant and/or magnetic permeability, there are more spaces for precise real-space manipulation for achieving topological photonic and phononic states^13–15^. The SOC which are required for realization of QSHE is either very small or hardly conceived in these systems, whereas the topological energy (or frequency) gap induced by the texture of hopping integral in a tight-bind picture is considerably large.

The present theoretical approach based on mirror winding number in TRS systems reveals the relation between the quantum pseudospin Hall effect, which mimics the quantum anomalous Hall effect and the quantum spin Hall effect and is defined in two dimensions (2D), and the Su-Schrieffer-Heeger (SSH) model^16^ and Rice-Mele model^17^ as the hallmark of 1D topology. This link is quite natural in the present 2D system, where the nontrivial topology arises from the bond alternating the same as the 1D counterpart. As a merit of this approach, we show that topologically protected edge states can be realized in terms of a variety of edge decorations at the graphene-vacuum interface in accordance to the bulk texture of hopping energy (see Fig. 1d).

## Results

### Model and Band Structure

As noted above, our model is a tight-binding Hamiltonian in honeycomb lattice $H={\sum }_{\langle ij\rangle }{t}_{ij}{c}_{i}^{\dagger }{c}_{j}$, where ${c}_{i}^{\dagger }$ (*c*_*i*_) is the creation (annihilation) operator at site *i*, and 〈*ij*〉 denotes summation over the nn sites, with the hopping integral *t*_*ij*_ modulated spatially (see also refs^18,19^). To be specific, we introduce two values of hopping energy, intra- and inter-hexagon as *t*_0_ and *t*_1_ (see Fig. 1a).

**Figure 2:**
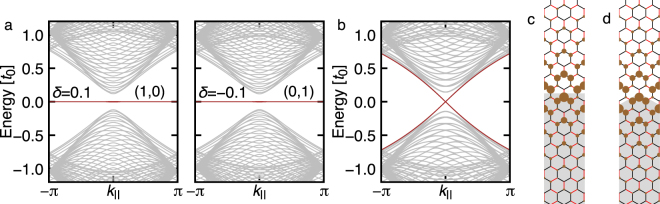
(**a**) The band structures for a ribbon of *graphene-zigzag* edge with masses of opposite signs. The edge modes are highlighted by the brown color. In calculations, the ribbon is long and periodic along ***a***_1_ and contains 40 unit cells along ***a***_2_. The mirror winding numbers (*n*_+_, *n*_−_) are also indicated. (**b**) The band structure for the interface between two regions *δ* = ±0.1 with *graphene-zigzag* type boundary. Calculations are performed using a system periodic along ***a***_1_ and consisting of repetition of 30 units of *δ* > 0 and *δ* < 0 cells in the perpendicular direction. (**c**) and (**d**) show a part of the system. For the black bonds, *t*_0_ = 1 is assigned throughout the system. For the red bonds, *t*_1_ = 0.9 (1.1) is assigned if a bond is in the white (gray) region. The hoppings across the two regions are set to the geometric mean of the corresponding hoppings in the two regions. (**c**) The weight of the interface state wave-function with *graphene-zigzag* type boundary shape. (**d**) The same for *molecule-zigzag* type boundary shape.

Let us begin with the numerically obtained band structures of a ribbon system with a simple zigzag edge (see Fig. 1c), which we name *graphene-zigzag* edge. Figure 2a shows the band structure as a function of the momentum parallel to the edge, *k*_||_. We clearly see a flat band edge mode regardless of the sign of *δ* = (*t*_1_ − *t*_0_)/*t*_0_, which is inherited from the zigzag-edged graphene without hopping texture^9^. That is, the edge mode is nothing like helical ones, although the bulk state acquires a gap due to the hopping modulation. Remarkably, putting two regions with *δ* > 0 and *δ* < 0 side by side, instead of the exposing to vacuum as in the ribbon geometry, the situation is drastically altered. Figure 2b shows the band structure for the system having the boundary between *δ* = ±0.1 with a *graphene-zigzag* interface. (See Figs 1d or 2c.) Now, the interface state becomes dispersive and counterpropagating, just like the helical edge modes in QSHE.

**Figure 3:**
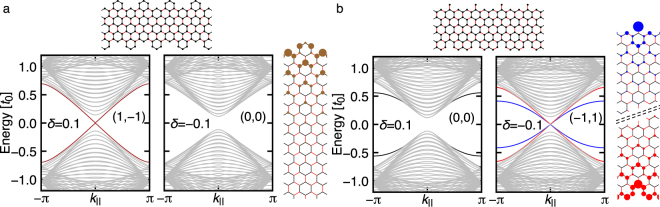
The band structures of ribbon systems for (**a**) *molecule-zigzag* and (**b**) *partially-bearded* edges. In (**a**) the counterpropagating edge states are highlighted by the brown dispersions for *δ* = 0.1 with double degeneracy, whereas in (**b**) the blue (red) dispersions are for the counterpropagating edge states localized at the top (bottom) edge. The weight of the wave function |*ψ*_*i*_|^2^ for the edge modes are displayed in the right most panels. The black lines in (**b**) for *δ* = 0.1 represent edge states whose origin is not topology. The computational setup is the same as Fig. 2a. The mirror winding numbers (*n*_+_, *n*_−_) are also indicated.

Interestingly, making an interface between *δ* positive and negative regions is not the only way to achieve counterpropagating topological modes. Appropriate decorations at the ribbon edge to vacuum also work well. Figure 3a illustrates a decoration and associated band structure for the ribbon, and clearly shows the counterpropagating edge mode for *δ* > 0 and no edge mode for *δ* < 0. We name the edge shape in Fig. 3a *molecule-zigzag* type, since it preserves hexagons formed by the bonds with *t*_0_, which are regarded as artificial molecules. Remarkably, we can think of a decoration leading to counterpropagating mode for *δ* < 0 instead of *δ* > 0. Such a decoration is illustrated in Fig. 3b with the corresponding band structure for the ribbon. We name the edge shape in Fig. 3b *partially-bearded* type, inspired by the structure at the top edge of the ribbon. In this case, the edge states at the top and bottom edges are different, due to the absence of symmetry with respect to the middle line of the ribbon.

With the clear edge-shape dependence of the edge mode observed in Fig. 3, one might feel curious on possible shape dependence of the interface states between the two regions with positive and negative *δ*. Our numerical analysis reveals that even if the boundary dividing the two regions is changed to *molecule-zigzag* or *partially-bearded* from the plain *graphene-zigzag*, the band structure remains almost unchanged from Fig. 2b. The wave functions for the interface state associated with the different boundary shapes are similar to each other (see Fig. 2c and d), signaling a weak influence of the interface shape on the topological interface states. (See also ref.^20^).

### Mirror Winding Number

So far, we have established that the topological modes at the ribbon edge depends on the edge shape, while the one at the interface between *δ* > 0 and *δ* < 0 regions does not. In the following, we provide a unified way of understanding this variety in terms of a topological invariant. Because the present system exhibits a bipartite nature, the Hamiltonian in *k*-space, which is obtained by the Fourier transformation from *c*_*i*_ to *c*_*k*_, can be written as

1 $${H}_{{\boldsymbol{k}}}=(\begin{array}{cc}0 & {Q}_{{\boldsymbol{k}}}\\ {Q}_{{\boldsymbol{k}}}^{\dagger } & 0\end{array}),$$

with a basis where the upper (lower) half is for A (B) sublattice and *Q*_***k***_ is a 3 × 3 matrix. The Hamiltonian (1) anticommutes with the chiral operator *γ* = diag(1, −1), manifesting the chiral symmetry (or sublattice symmetry) of the present system. Then, regarding the momentum ***k*** parallel to the unit vector ***a***_1_ defined in Fig. 1a as a free parameter, the system can be viewed as an effective 1D model, to which one can assign the winding number supported by the chiral symmetry as^21^,

2 $$n({k}_{||})=-\frac{1}{2\pi }{\int }_{0}^{2\pi }\frac{d}{d{k}_{\perp }}{\rm{\arg }}\,({\rm{\det }}\,{Q}_{{k}_{\perp },{k}_{||}})d{k}_{\perp }\mathrm{.}$$

For *k*_||_ = 0, we can do further. That is, the mirror symmetry with the mirror plane perpendicular to ***a***_1_ enables us to decompose the Hamiltonian (1) into even and odd sectors ${H}_{{k}_{\perp }}^{\pm }$, where ***k*** is replaced by *k*_⊥_, since our focus is now on *k*_||_ = 0. In this case, the mirror operation commutes with *γ*, and thus, *Q*_***k***_ can be decomposed into even and odd sectors ${Q}_{{{\boldsymbol{k}}}_{\perp }}^{\pm }$. Then, just as first pointed out in ref.^22^ (and also discussed in the following papers^23–26^), we can assign winding numbers for the even and odd sectors separately by plugging ${Q}_{{{\boldsymbol{k}}}_{\perp }}^{\pm }$ into equation (2), which constitutes the *mirror* winding number (*n*_+_, *n*_−_). Note that even though we also have a mirror plane parallel to ***a***_1_, which is related to *armchair* type edge, another important class of graphene edge, that mirror operation does not commute with *γ*, leading to no mirror winding number for *armchair* edge. Note also that in a general context, the approach intertwining topology and crystal symmetry was first considered in the work on topological crystalline insulator^27,28^.

**Figure 4:**
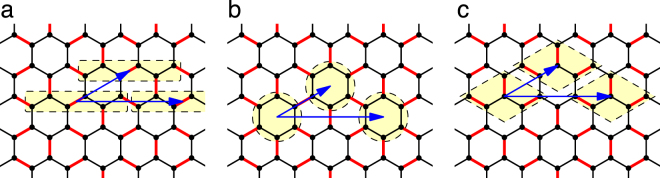
Unit cells leading to (**a**) *graphene-zigzag*, (**b**) *molecule-zigzag*, and (**c**) *partially-bearded* edges.

In general, a winding number depends on the choice of unit cell^29,30^. Although the choice of unit cell is merely a unitary transformation of *H*_***k***_, it does not necessarily correspond to a unitary transformation of *Q*_***k***_. Consequently, the unit cell choice can modify the winding number through equation (2). It is known that in order to make one-to-one correspondence between edge modes and the winding number, we must obey a rule such that a chosen unit cell is not chopped by the considered edge^30^. Namely, if the edge shape is changed, the unit cell should be changed correspondingly. In the present case, three kinds of unit cells obeying this rule for *graphene-zigzag*, *molecule-zigzag*, and *partially-bearded* edges are given in Fig. 4.

Equipped with the appropriate unit cell, *Q*_***k***_ for *graphene-zigzag* edge becomes

3 $${Q}_{{\boldsymbol{k}}}=(\begin{array}{ccc}{t}_{1}X\bar{Y} & {t}_{0} & {t}_{0}\\ {t}_{0} & {t}_{1}\bar{X} & {t}_{0}\bar{Y}\\ {t}_{0} & {t}_{0}\bar{Y} & {t}_{1}\end{array}),$$

where $X={e}^{i{\boldsymbol{k}}\cdot {{\boldsymbol{a}}}_{1}}$ and $Y={e}^{i{\boldsymbol{k}}\cdot {{\boldsymbol{a}}}_{2}}$. At *k*_||_ = 0 (*X* = 1), *Q*_***k***_ is decomposed into

4 $${Q}_{{k}_{\perp }}^{+}=(\begin{array}{cc}{t}_{1}\bar{Y} & \sqrt{2}{t}_{0}\\ \sqrt{2}{t}_{0} & {t}_{1}+{t}_{0}\bar{Y}\end{array}),\,{Q}_{{k}_{\perp }}^{-}={t}_{1}-{t}_{0}\bar{Y},$$

which leads to (*n*_+_, *n*_−_) = (1, 0) for *δ* > 0 and (0,1) for *δ* < 0. On the other hand, for *molecule-zigzag* edge, we have

5 $${Q}_{{\boldsymbol{k}}}=(\begin{array}{ccc}{t}_{1}X{\bar{Y}}^{2} & {t}_{0} & {t}_{0}\\ {t}_{0} & {t}_{1}\bar{X}Y & {t}_{0}\\ {t}_{0} & {t}_{0} & {t}_{1}Y\end{array})$$

and

6 $${Q}_{{k}_{\perp }}^{+}=(\begin{array}{cc}{t}_{1}{\bar{Y}}^{2} & \sqrt{2}{t}_{0}\\ \sqrt{2}{t}_{0} & {t}_{0}+{t}_{1}Y\end{array}),\,{Q}_{{k}_{\perp }}^{-}={t}_{1}Y-{t}_{0},$$

leading to (*n*_+_, *n*_−_) = (1, −1) for *δ* > 0 and (0, 0) for *δ* < 0. The same prescription applies to *partially-bearded* edge, giving us

7 $${Q}_{{\boldsymbol{k}}}=(\begin{array}{ccc}{t}_{1} & {t}_{0} & {t}_{0}\\ {t}_{0}Y & {t}_{1} & {t}_{0}X\bar{Y}\\ {t}_{0}\bar{X}Y & {t}_{0}\bar{Y} & {t}_{1}\end{array}),$$

and

8 $${Q}_{{k}_{\perp }}^{+}=(\begin{array}{cc}{t}_{1} & \sqrt{2}{t}_{0}\\ \sqrt{2}{t}_{0}Y & {t}_{1}+{t}_{0}\bar{Y}\end{array}),\,{Q}_{{k}_{\perp }}^{-}={t}_{1}-{t}_{0}\bar{Y},$$

which leads to (*n*_+_, *n*_−_) = (0, 0) for *δ* > 0 and (−1, 1) for *δ* < 0. The results are summarized in Table 1. With the mirror winding numbers one can easily obtain the total winding number as *n*_tot_ = *n*_+_ + *n*_−_. While the mirror winding number is only defined at *k*_||_ = 0, the total winding number *n*_tot_ is well defined for any momentum, and is conserved as far as the bulk gap is not closed. It is worthy noticing that, as can be read from Table 1, the states for *δ* > 0 and *δ* < 0 can be distinguished topologically in terms of the mirror winding numbers, but indistinguishable by the total winding number.

**Table 1:** Mirror winding number (*n*_+_, *n*_−_) at *k*_||_ = 0 for three edge shapes.

(*n*_+,_ *n*_−_)	*δ* > 0	*δ* > 0
*molecule-zigzag*	(1, −1)	(0, 0)
*partially-bearded*	(0, 0)	(−1, 1)
*graphene-zigzag*	(1, 0)	(0, 1)

Now let us see the correspondence between the mirror winding number (Table 1) and the edge modes (Figs 2 and 3). For *graphene-zigzag* edge, *n*_tot_ = 1 for any *k*_||_ regardless of the sign of *δ*. Because vacuum is topologically trivial with zero mirror winding number, we expect the flat-band edge mode associated with the the nonzero total winding number^16,17,29^ for the ribbon geometry, which is in accordance with Fig. 2a. On the other hand, for *molecule-zigzag* and *partially-bearded* edges, we observe the counterpropagating edge modes with *n*_±_ ≠ 0 and *n*_tot_ = 0. This can be understood as follows: for *k*_||_ = 0, one has two zero-energy edge modes, one from the even sector and the other from the odd sector; for *k*_||_ ≠ 0, there is no reason to have any zero-energy edge mode since *n*_tot_ = 0. As the consequence, we end up with two edge modes with finite dispersions and crossing linearly at *k*_||_ = 0. Remarkably, the counterpropagating modes at the *graphene-zigzag* interface between two domains with opposite values of *δ* (Fig. 2b) admits exactly the same explanation, where one takes the differences of mirror winding numbers between the two domains. In this way, all the results in Figs 2 and 3 are explained in a unified manner by means of the mirror winding number.

### Analytic Solutions for the Wave Functions

**Figure 5:**
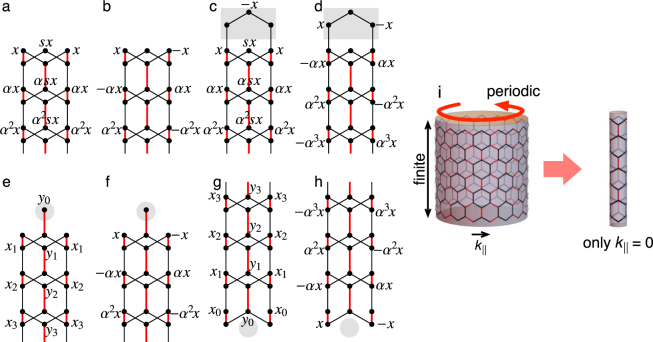
Schematics of the ansatz for the effective 1D model at *k*_||_ = 0: (**a**,**b**) for *graphene-zigzag* edge, (**c**,**d**) for *molecule-zigzag* edge, (**e**,**f**)/(**g**,**h**) for the top/bottom edge of *partially-bearded* edge. (**a**,**c**,**e**,**g**)/(**b**,**d**,**f**,**h**) are for the solutions of even/odd parity with respect to the mirror operation. The shaded regions for *graphene-zigzag* and *partially-bearded* edges can be regarded as decorations to *graphene-zigzag* edge. (**i**) The 1D effective model can be considered as a model on a cylinder with the smallest diameter, where *k*_||_ = 0 is the only allowed momentum.

A more intuitive and physical understanding of this unified picture is available by checking analytic solutions of the wave functions for the topological edge states. Here, we briefly describe the derivation of the wave functions for the zero-energy topological edge modes at *k*_||_ = 0, where the mirror symmetry is effective with which solutions are classified into even- and odd-parity ones. For the study of edge modes, one often puts the system on a cylinder, open in one direction and periodic in the other direction (see Fig. 5). We can obtain *k*_||_ dependence of the edge band structure in the large diameter limit. In contrast, in the small diameter limit, *k*_||_ = 0 is the only allowed momentum along the edge, which is regarded as an effective one-dimensional model for *k*_||_ = 0. Figure 5 shows those effective 1D models, where (a) and (b) correspond to the *graphene-zigzag* edge, (c) and (d) to the *molecule-zigzag* edge, and (e-h) to the *partially-bearded* edge, respectively. The chiral symmetry permits us to adopt the following ansatz for the wave functions of zero-energy edge modes Ψ_*A*_ = (*ψ*_*A*_, 0) or Ψ_*B*_ = (0, *ψ*_*B*_).

Let us begin with the even-parity solution for the *graphene-zigzag* edge (Fig. 5a). From the second site on the right (or left) column, one requires (*s* + *α*)*t*_0_ + *t*_1_ = 0^31,32^ for a nontrivial zero-energy mode. From the fourth site on the central column, one has 2*t*_0_ + *αst*_1_ = 0. These two equations are satisfied by $\alpha =-\beta +\sqrt{{\beta }^{2}+\mathrm{1/}\beta }$ and $s=-\beta -\sqrt{{\beta }^{2}+\mathrm{1/}\beta }$ with *β* ≡ (1 + *δ*)/2. The solution is physically relevant only when it decays from the edge into the bulk, namely when *α* < 1, which is achieved by *δ* > 0. On the other hand, for the odd-parity solution (Fig. 5b), the ansatz gives a solution with *α* = 1 + *δ*, which becomes physical when *δ* < 0. These results are in accordance with Table 1, namely (*n*_+_, *n*_−_) = (1, 0) for *δ* > 0 while (*n*_+_, *n*_−_) = (0, 1) for *δ* < 0 for the *graphene-zigzag* edge.

Next, we move on to the *molecule-zigzag* edge (Fig. 5c and d). Comparing Fig. 5a and c, we notice that the even-parity solution for the *molecule-zigzag* edge is essentially unchanged from the one for the *graphene-zigzag* edge, where one has a physical solution when *δ* > 0. On the other hand, for the odd-parity solution (Fig. 5d), the ansatz gives a solution with *α* = 1/(1 + *δ*), which is physical for *δ* > 0. That is, the even- and odd-parity solutions are both physical when and only when *δ* > 0, in agreement with (*n*_+_, *n*_−_) in Table 1.

For the *partially-bearded* edge, the top and the bottom edges should be considered separately since the ribbon becomes asymmetric with respect to the middle line of the ribbon (see Fig. 3b). A comparison between Fig. 5b,f, and h tells us that the odd-parity solutions are essentially the same as those in the *graphene-zigzag* edge. [Note that the figure for the bottom edge, Fig. 5h, is upside-down.] Therefore, we have a physical solution for *δ* < 0. On the other hand, the even-parity solutions are different from those for the *graphene-zigzag* edge. For both of the top (Fig. 5e) and the bottom (Fig. 5g) edges, the parameters *x*_*i*_ and *y*_*i*_ satisfy the relation

9 $$(\begin{array}{c}{x}_{j+1}\\ {y}_{j+1}\end{array})=A(\begin{array}{c}{x}_{j}\\ {y}_{j}\end{array}),\quad A=(\begin{array}{cc}0 & -\beta \\ -1 & 2{\beta }^{2}\end{array})\mathrm{.}$$

The solution becomes physical when the absolute values of the two eigenvalues of *A* get smaller than unity, which is achieved by *δ* < 0. Again, these results are in accordance with Table 1, i.e., two physical solutions (even and odd) for *δ* < 0 at the *partially-bearded* edge.

Now, let us interpret the results starting from the *graphene-zigzag* ribbon. Noting that *molecule-zigzag* and *partially-bearded* edges are obtained by some appropriate decoration, i.e., adding or subtracting a few sites at the edge. Then, by examining the wave functions in ribbons given in Fig. 5, it turns out that the decoration leading to *molecule-zigzag* edge affects only the mirror-odd sector, while the decoration leading to *partially-bearded* edge affects only the mirror-even sector. As we have discussed, the unit cell must be adapted when the edge shape is modified (Fig. 4), influencing the winding number. In our case, the change from the graphene-zigzag to the molecule-zigzag (partially-bearded) reduces the mirror winding number for the odd (even) sector by 1, which explains Table 1. To summarize, the selective action on either of mirror-even or odd sector is the key to observe interesting evolutions of the topological edge modes.

## Discussions

In the previous work^8^, the parameter regime *t*_1_ < *t*_0_ was regarded as topologically trivial. However, as summarized in Table I this parameter regime supports a state characterized by nonzero mirror winding numbers when the rhombic unit cell is chosen (see Fig. 4), instead of the circular one presumed in the previous work. This is a unique feature of the present approach for achieving topological states in terms of real-space modulation. On one hand, a relation between the unit cell and the underlying hopping texture determines the explicit form of the Hamiltonian [see equations (3–8)]. As a result, the unit cell choice affects the winding number. On the other hand, the unit cell determines the edge shape in our approach. In this sense, topology (winding number), real-space textures, and edge shapes are related, which is useful for designing topological edge states. Such a designability possibly leads to switch and diode^33,34^ utilizing topological edge states by dynamically controlling edge location and shape with local gate voltages.

In our theory, the edge shape plays an essential role. For any kinds of artificial realization of the textured honeycomb model^11,13–15^, the edge shape is also likely to be artificially modified, and our theory is immediately in effect. In addition, we would like to point out that the atomically precise control over the edge shape of a graphene ribbon is a developing experimental technique^35–37^.

To summarize, topological phases in honeycomb lattice induced by texture in hopping energy between nearest-neighboring sites are characterized in terms of the mirror winding number. Analytic wave functions are provided for zero-energy edge modes at the Γ point, which evolve into the dispersive counterpropagating edge states at finite momenta. Explicitly, when the intra-hexagon hopping energy is stronger (weaker) than the inter-hexagon one, *molecule-zigzag* (*partially-bearded*) edge yields gapless counterpropagating edge modes. The present work provides a new designing guideline for topological edge states adaptive to the bulk hopping texture, which may pave a way to tailoring graphene and related materials in the topological point of view.

### Data availability statement

The datasets generated during the current study are available from the corresponding author on reasonable request.
